# Supplementary material for: Automated Mucormycosis Diagnosis from Paranasal CT Using ResNet50 and ConvNeXt Small
Source: Bioengineering (Basel). 2025 Aug 8;12(8):854. doi: 10.3390/bioengineering12080854 (PMC12383388; doi:10.3390/bioengineering12080854)
Supplement: Supplementary file 1 [file bioengineering-12-00854-s001.zip › bioengineering-3752149 - Supplementary.pdf]

bioengineering

MDPI

Article

Automated Mucormycosis Diagnosis from Paranasal CT Using ResNet50 and ConvNeXt Small

Serdar Ferit Toprak [1], Serkan Dedeoğlu [2,*], Günay Kozan [3], Muhammed Ayral [3], Şermin Can [3], Ömer Türk [4] and Mehmet Akdağ [3]

[1] Department of Audiology, Artuklu University, Mardin, 47100, Turkey; serdarferit@yahoo.com
[2] Department of Otorhinolaryngology, University of Health Sciences Gazi Yasargil Training and Research Hospital, Diyarbakır, 21100, Turkey; drserkandedeoglu@gmail.com (S. D.)
[3] Department of Otorhinolaryngology and Head and Neck Surgery Clinic, Dicle University Faculty of Medicine, Diyarbakir, 21010, Turkey; gunaykozan@hotmail.com (G.K.); drayral@hotmail.com (Muhammed Ayral); sermin.can@hotmail.com (Ş.C.); mehmet.akdag@dicle.edu.tr (Mehmet Akdağ)
[4] Department of Computer Engineering, Faculty of Engineering and Architecture, Mardin Artuklu University, Mardin, 47100, Turkey; omerturk@artuklu.edu.tr
* Correspondence: drserkandedeoglu@gmail.com; Tel: +9005325999729

Table S1. 5-fold cross-validation results per fold (accuracy ± SD) for ResNet50 and ConvNeXt.

Fold	Model	Accuracy (%)	Precision	Recall	F1-score
1	ConvNeXt-Small	100.0	1.00	1.00	1.00
	ResNet50	98.3	0.98	0.98	0.98
2	ConvNeXt-Small	100.0	1.00	1.00	1.00
	ResNet50	97.9	0.98	0.98	0.98
3	ConvNeXt-Small	97.5	0.98	0.98	0.98
	ResNet50	97.1	0.97	0.97	0.97
4	ConvNeXt-Small	100.0	1.00	1.00	1.00
	ResNet50	96.8	0.97	0.97	0.97
5	ConvNeXt-Small	100.0	1.00	1.00	1.00
	ResNet50	97.5	0.97	0.97	0.97
**Avg ± SD**	ConvNeXt-Small	**98.9 ± 1.1**	**0.99 ± 0.01**	**0.99 ± 0.01**	**0.99 ± 0.01**
	ResNet50	**97.5 ± 0.6**	**0.97 ± 0.01**	**0.97 ± 0.01**	**0.97 ± 0.01**

Table S2. 5-Fold Cross-Validation Confusion Counts for Each Model

Fold	Model	True Normal	False Normal→Polyp	False Normal→Mucor	True Polyp	False Polyp→Normal	False Polyp→Mucor	True Mucor	False Mucor→Normal	False Mucor→Polyp
1	ConvNeXt	17	0	0	23	0	0	12	0	0
	ResNet50	17	0	0	23	0	0	12	0	0

Fold	Model	True Normal	False Normal→Polyp	False Normal→Mucor	True Polyp	False Polyp→Normal	False Polyp→Mucor	True Mucor	False Mucor→Normal	False Mucor→Polyp
2	ConvNeXt	18	0	0	22	0	0	12	0	0
	ResNet50	18	0	0	22	0	0	12	0	0
3	ConvNeXt	17	0	0	22	0	0	13	0	0
	ResNet50	17	0	0	22	0	0	13	0	0
4	ConvNeXt	16	0	0	23	0	0	13	0	0
	ResNet50	16	0	0	23	0	0	13	0	0
5	ConvNeXt	16	0	0	23	0	0	13	0	0
	ResNet50	15	0	0	22	1	0	13	0	0

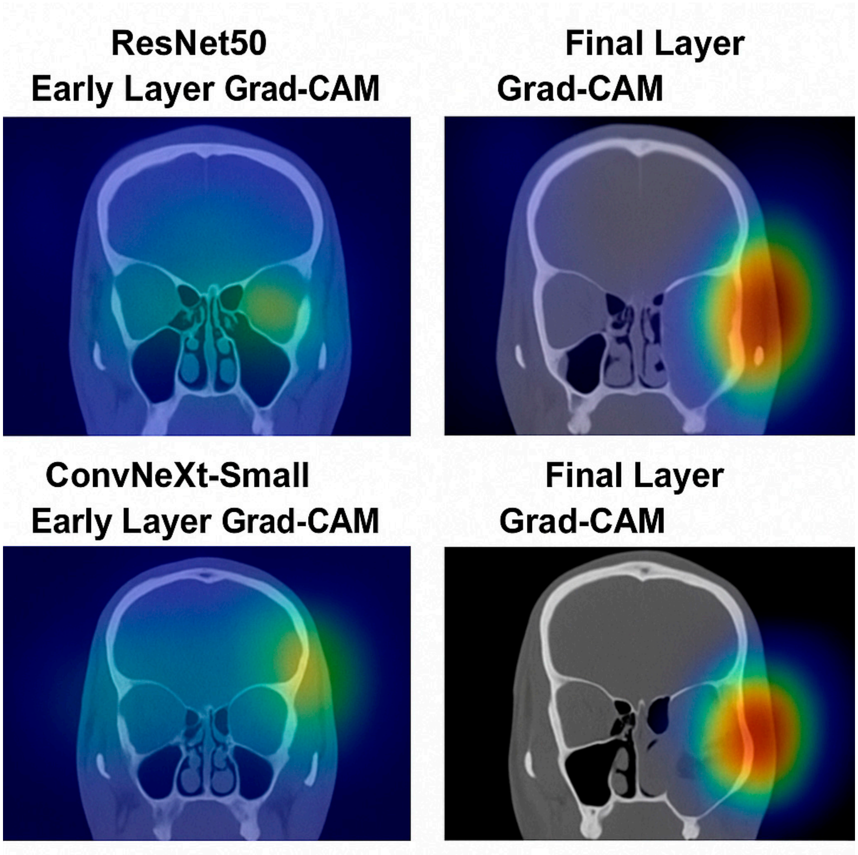

Figure S1: Grad-CAM maps for early vs. late layers of each model.
